# Isolated mycobiota from carcasses of wild animals run over on Highway 493 in Seropédica, Rio de Janeiro, Brazil

**DOI:** 10.29374/2527-2179.bjvm006425

**Published:** 2026-06-19

**Authors:** Clarissa Angélica Azevedo Pardão, Jacques Fideles da Silva, Júlia Aline Santos de Mello Pereira, Mário Mendes Bonci, Carlos Alexandre Rey Matias, Águida Aparecida de Oliveira

**Affiliations:** 1 Instituto de Veterinária, Universidade Federal Rural do Rio de Janeiro, Seropédica, RJ, Brazil; 2 Instituto de Florestas, Universidade Federal Rural do Rio de Janeiro, Seropédica, RJ, Brazil; 3 Departamento de Microbiologia e Imunologia, Instituto de Veterinária, Universidade Federal Rural do Rio de Janeiro, Seropédica, RJ, Brazil; 4 Departamento de Epidemiologia e Saúde Pública, Instituto de Veterinária, Universidade Federal Rural do Rio de Janeiro, RJ, Brazil.

**Keywords:** fungus, cadaveric decomposition, roadkill, environmental impact, ecosystem, fungos, decomposição cadavérica, atropelamento de fauna, impacto ambiental, ecossistema

## Abstract

The ''barrier effect'' is caused by the intense construction of highways in the present day. It’s a phenomenon that modifies the dynamics of circulation and disperses animal communities, isolating them on both sides of the highways and reducing the gene flow of the population. In order to evaluate its impact, over a period of two years, the monitoring of the wild fauna run over on a federal highway in Seropédica, Rio de Janeiro, Brazil was carried out. Samples of animals found dead were identified and sown on mycological culture media, and the fungi were later identified. The three most frequently found animal species were, respectively: bat (Ordem Chiroptera), opossum (*Didelphis aurita*), and capybara (*Hidrochoerus hidrochaeris*). A great diversity of fungi was found in all the 55 samples of cadaveric remains from the carcasses found throughout the period – different species belonging to more than 10 fungal genera, including *Mucor* sp. and *Rhyzopus* sp*.* (belonging to the Order Mucorales), *Aspergillus* sp., *Acremonium* sp., *Cladosporium* sp. and *Fusarium* sp. (These last 4 belong to the phylum Ascomycota). This study solidifies the understanding about the impacts caused by anthropic expansion on Brazilian fauna and the succession of mycoobiota in cadaveric decomposition.

## Resumo

O “efeito barreira” é causado pela intensa construção de rodovias atualmente. Trata-se de um fenômeno que modifica a dinâmica de circulação e dispersão das comunidades animais, isolando-as em ambos os lados das rodovias e reduzindo o fluxo gênico entre as populações. Com o objetivo de avaliar esse impacto, foi realizado, ao longo de dois anos, o monitoramento da fauna silvestre atropelada em uma rodovia federal no município de Seropédica, no Rio de Janeiro, Brasil. Amostras de animais encontrados mortos foram identificadas e semeadas em meios de cultura micológicos, e os fungos foram posteriormente identificados. As três espécies animais mais frequentemente encontradas foram, respectivamente: morcegos (ordem Chiroptera), gambás (*Didelphis aurita*) e capivaras (*Hydrochoerus hydrochaeris*). Uma grande diversidade de fungos foi encontrada em todas as 55 amostras de restos cadavéricos provenientes das carcaças coletadas ao longo do período do estudo, incluindo diferentes espécies pertencentes a mais de 10 gêneros fúngicos, como *Mucor* sp. e *Rhizopus* sp. (pertencentes à ordem Mucorales), *Aspergillus* sp., *Acremonium* sp., *Cladosporium* sp. e *Fusarium* sp. (estes quatro últimos pertencentes ao filo Ascomycota). Este estudo contribui para o entendimento dos impactos da expansão antrópica sobre a fauna brasileira e sobre a sucessão da micobiota durante a decomposição cadavérica.

## Introduction

The Brazilian Road system, which extends widely through the Atlantic Forest, is a key element for the country's logistics and economic development (Freitas et al., 2010). However, its vast network creates the so-called "barrier effect", a phenomenon that alters circulation, isolates animal populations on either side of highways, reduces gene flow, and increases the risk of extinction. Highways also favor the introduction of exotic species and greater human access to natural habitats, facilitating wildlife hunting (Milli & Passamani, 2006). Many animals cross these barriers daily and are run over while attempting to do so.

The Atlantic Forest shelters numerous endangered species, essential for ecosystem preservation, and is considered a global hotspot, along with 33 other regions (Pinto et al., 2006). Thus, Brazil’s extensive road network poses an additional threat to fauna, both by deforestation for construction and through wildlife-vehicle collisions. Near Seropédica, Rio de Janeiro, the BR-465 highway borders the Mário Xavier National Forest, a 495-hectare Conservation Unit managed by ICMBio and home to critically endangered species such as *Physalaemus soaresi* and *Notolebias minimus* (Instituto Chico Mendes de Conservação da Biodiversidade, 2022). Roadkill numbers are underestimated, as carcasses are often removed by people or scavengers, and large animals may not die at the collision site (Faria et al., 2022). Frequent collisions, for example, involving *Physalaemus soaresi*, can have irreversible consequences, as seen with *Tapirus terrestris* in Morro do Diabo State Park (Medici et al., 2012). The heavy traffic of vehicles and the lack of safe structures for wildlife crossings have a significant negative impact on the region (Instituto Chico Mendes de Conservação da Biodiversidade, 2022). Along roadsides, carcasses in different stages of decomposition are common and represent valuable material for study.

Identifying roadkill animals and subsequently identifying the mycobiota on the carcasses helps in understanding the impact of urbanization on wildlife, while also contributing to studies in forensic mycology. Fungi are particularly relevant due to their ability to survive in varied substrates. Wild animals, both free-ranging and captive, are susceptible to mycoses. Environmental fungi are saprophytes usually found in soil, vegetation, seeds, and grains. Dermatophytoses are superficial mycoses affecting skin and hair, impacting both humans and animals, with easy cross-species transmission Animals may act as subclinical carriers, developing lesions under stress, malnutrition, or illness, since these fungi are opportunistic. While *Microsporum canis* and *Trichophyton* spp. are considered primary dermatophytes rather than opportunistic fungi, stress, malnutrition, or underlying disease may increase susceptibility and facilitate the development of lesions (Chaves et al., 2021; De Hoog et al., 2021). Wild and exotic animals can also be affected, such as rabbits infected by *Trichophyton mentagrophytes* (Franklin et al., 1991). Although rare, dermatophytosis has been reported in reptiles as well (Rosen, 2011).

Few studies address which fungi are present during cadaveric decomposition in animals and humans, and most use molecular biology. (Moitas et al., 2024; Tranchida et al, 2025; Zhao et al., 2024). Investigating the mycobiota of wild animals and their carcasses is essential, as these fungi provide insights into pathogenesis, species profiling, anthropogenic impacts, and fungal dynamics during decomposition Such studies also allow comparisons with the mycobiota of free-ranging and captive animals. These investigations are valuable for forensic science, especially in cases lacking entomological evidence. In addition to contributing to the assessment of the impact of urbanization on wildlife, this study also used decomposing materials from different species to investigate the effect of residual mycobiota on these carcasses. This article aims to determine the frequency of mycobiota on carcasses remains of wild animals found run over on a federal highway in Seropédica, Rio de Janeiro, Brazil.

## Material and methods

**Figure 1:**
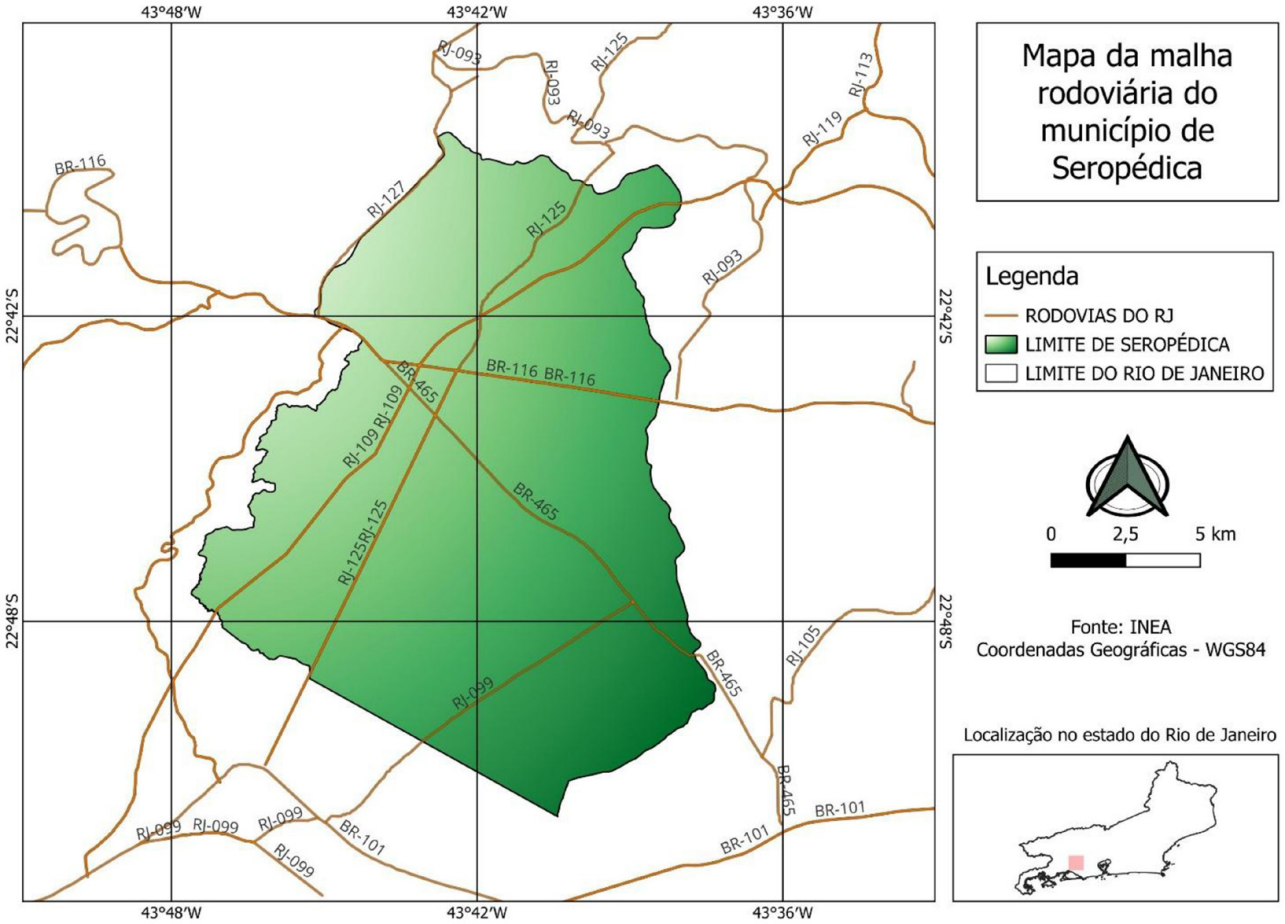
Map of the road network of the municipality of Seropédica, Rio de Janeiro, Brazil, highlighting the BR-465. Spatial distribution of the main federal (BR) and state (RJ) highways that cross the municipality of Seropédica, highlighting the BR- 465 - the section where this study was developed. The map shows the municipal boundary (in green), the state boundary of Rio de Janeiro (in gray), graphic scale, cartographic orientation, and location of Seropédica within the state of Rio de Janeiro. WGS84 geodetic reference system. Cartographic data from the State Environmental Institute (INEA).

Over a two-year period (2022–2024), weekly monitoring of wildlife roadkill was conducted along a ~20 km stretch of the BR-493 highway (Brazil) (Figure 1), also known as Arco Metropolitano road, between the Seropédica (state of Rio de Janeiro) access point and the return loop toward the municipality in the direction of Itaguaí (state of Rio de Janeiro). Monitoring was carried out using a passenger vehicle traveling at approximately 50 km/h (half the maximum speed limit on the highway). If roadkilled animals were detected, their data was recorded.

**Figure 2:**
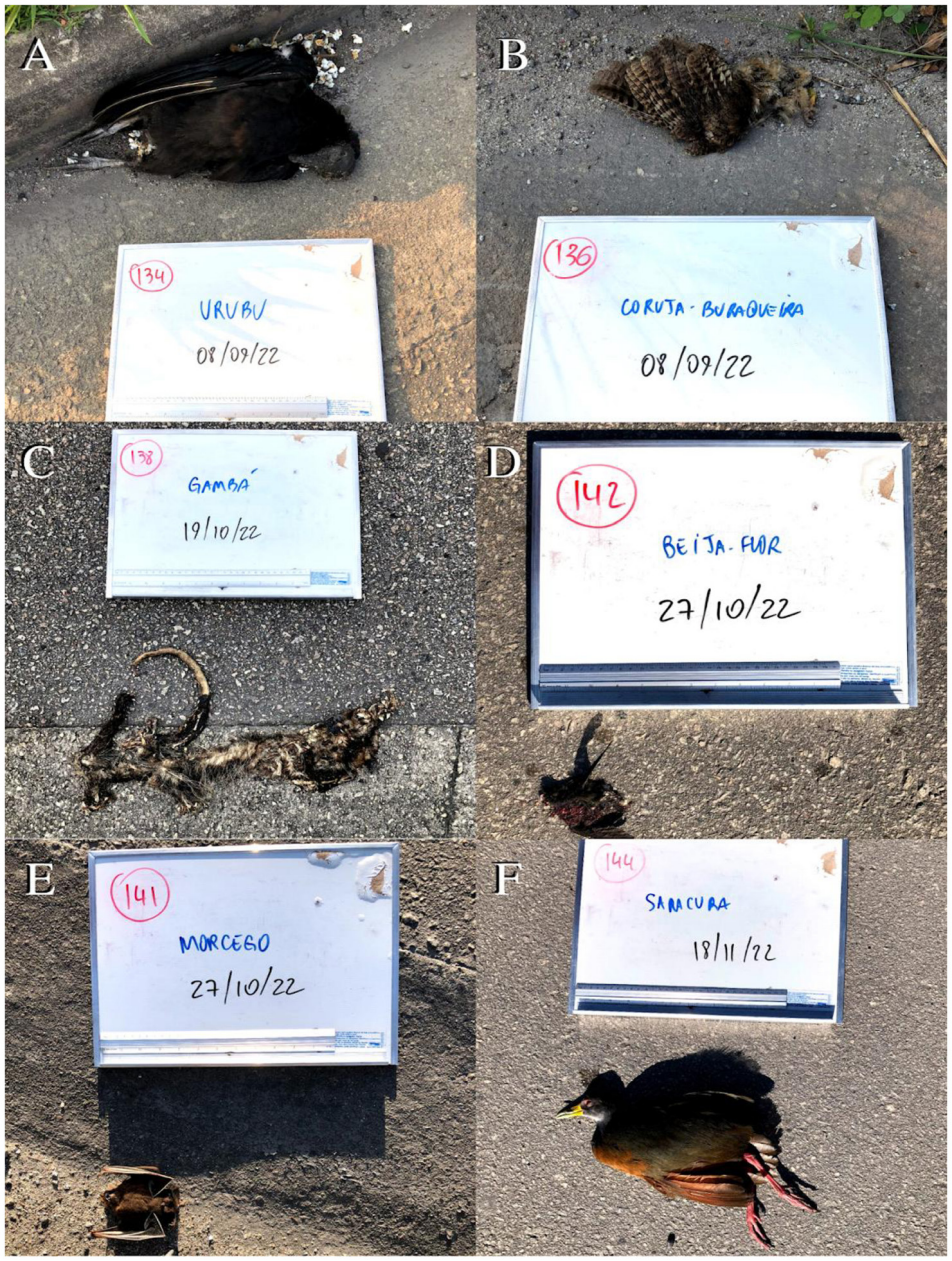
Carcasses of animals recovered along the BR-493 stretch. A – Vulture black (*Coragyps atratus*), B – Burrowing Owl, C – Opossum (*Didelphis aurita*), D – Hummingbird (*Eupetomena* sp.), E – Bat (Chiroptera), F – Yellow-legged Rail (*Aramides cajaneus*).

When the carcass of a wild animal (amphibian, reptile, bird, or mammal)—was identified, the site was isolated for species identification; assessment of conservation status at both national and regional levels; photographic documentation; geographic coordinate recording; and collection of fur, scales, or feathers using gloves to avoid contamination. All carcasses were in a state of skeletonization or advanced decomposition (Figure 2). The carcasses were collected in sterile paper envelopes until processing. All data collection was carried out using appropriate PPE.

Samples of animals found dead were identified and sown on Sabouraud Agar added with chloramphenicol and on Mycosel® Agar (Biolog) (cycloheximide and antibiotic) for 21 days at 28 °C. After fungal growth, microscope slides were prepared using a drop of lactophenol cotton blue stain for hyaline (light-colored) fungi, and a drop of clearing agent (KOH 20%) for dematiaceous (dark-colored) fungi. Prepared slides were examined under a light microscope at 400× magnification. The fungi were identified solely at the phenotypic level. In cases where colony growth suggested *Fusarium* sp., or when only sterile hyphae (without conidia or macroconidia) were observed, the samples were subcultured on PDA (Potato Dextrose Agar) or MEA (Malt Extract Agar), as described by Sidrim & Rocha (2004) and De Hoog et al., 2021. Throughout the cultivation process, new isolations were performed, always with the aim of obtaining pure colonies.

For the identification of the fungi grown on the plates and on the manes, the following characteristics were observed: growth characteristics of the colonies, such as color and appearance (macromorphology) and characteristics of mycelium, presence, shape, size and septation of macroconidia; abundance and roughness of microconidia; presence or absence of chlamydoconidium; presence or absence of forms of sexual reproduction; hyphal septation (Samson et al., 2019; Sidrim & Rocha, 2004, De Hoog et al., 2021).

## Results

Of the 55 animals identified, 30 were mammals, 19 were birds, 3 were reptiles, and 3 were amphibians (Table 1). Based on the methodology adopted, the results regarding the mycobiota over a two years period are presented below, as shown in Table 2. Among the filamentous fungi identified (Table 2), the following distribution was observed: *Aspergillus* sp. (n = 15), *Fusarium* sp. (n = 8), *Cladosporium* sp. (n = 4), *Rhizopus* sp. (n = 2), *Curvularia* sp. (n = 4), *Chrysonilia* sp. (n = 2), *Basidiobolus* sp. (n = 1), *Nigrospora* sp. (n = 1), *Fonsecaea* sp. (n = 1), *Mucor* sp. (n = 1), *Pestalotia* sp. (n = 1), sterile hyphae (n = 1), other dematiaceous fungi (n = 5), and other Mucorales (n = 15). Fungi from the order Mucorales—characterized by rapid growth and coenocytic hyphae—are typically saprophytic organisms (De Hoog et al., 2021). In this study, species from this order were identified in association with cadaveric decomposition of animal carcasses, reinforcing their role not only as environmental decomposers of plant matter but also as key agents in the post-mortem microbial succession in wildlife.

**Table 1:** Geographic coordinates of the locations where wild animals were found (Seropédica – Rio de Janeiro).

**Sample**	**Animal**	**Taxonomy**	**Coordinates**
1	Opossum	*Didelphis aurita*	X -22.72702; Y -43.71387
2	Bat	Order Chiroptera (Molossidae)	X -22.71319; Y -43.70353
3	Common Waxbill	*Estrilda astrild*	X -22.73198; Y -43.71690
4	Crab-eating Raccoon	*Procyon cancrivorus*	X -22.73613; Y -43.71932
5	Bat	Order Chiroptera (Phyllostomidae)	X -22.74200; Y -43.72262
6	Capybara	*Hydrochoerus hydrochaeris*	X -22.69147; Y -43.68506
7	Bat	Order Chiroptera (Phyllostomidae)	X -22.71626; -43.70629
8	Bat	Order Chiroptera (Phyllostomidae)	X -22.73564; Y -43.71890
9	Caracara	*Caracara plancus*	X -22.74749; Y -43.72513
10	Crab-eating Fox	*Cerdocyon thous*	
11	Capybara	*Hydrochoerus hydrochaeris*	X -22.70195; Y -43.69527
12	Black Vulture	*Coragyps atratus*	X -22.69987; Y -43.69387;
13	Burrowing Owl	*Athene cunicularia*	X -22.72637; Y -43.71300
14	Opossum	*Didelphis aurita*	X -22.70465; Y -43.69729
15	Bat	Order Chiroptera (Phyllostomidae)	
16	Bat	Order Chiroptera (Phyllostomidae)	X -22.74676; Y -43.72511
17	Hummingbird	Family Trochilidae	X -22.72652; Y -43.71313;
18	Bat	Order Chiroptera (Phyllostomidae)	X -22.75600; Y43.73098
19	Yellow-legged Rail	*Aramides cajanea*	X -22.74313; Y -43.72285
20	Butter Frog	*Leptodactylus latrans*	X -22.70865; Y-43.69934
21	Snake	Ophidia	X -22.70475; Y -4369735
22	Eastern Screech Owl	*Megascops choliba*	X -22.72770; Y -43.71444
23	Opossum	*Didelphis aurita*	X -22.72273; Y -43.71043
24	Crab-eating Fox	*Cerdocyon thous*	X -22.73651; Y -43.71936
25	Crab-eating Fox	*Cerdocyon thous*	X -22.73083; Y -43.71618
26	Capybara	*Hydrochoerus hydrochaeris*	X -2271139; Y -43.70118
27	Opossum	*Didelphis aurita*	X -22.75203; Y -43.72750
28	Neotropical porcupine	Family Erethizontidae	X -22.70325; Y -43.69642
29	Marmoset	*Callithrix* sp.	X -22.72550; Y -43.71286
30	Opossum	*Didelphis aurita*	X -22.72930; Y-43.71507
31	Crab-eating Raccoon	*Procyon cancrivorus*	X-22.69400; Y-43.68803
32	Neotropical porcupine	Erethizontidae	X-22.72704; Y-43.71352
33	Opossum	*Didelphis aurita*	X-22.72671; Y-43.71374
34	Black Curassow	*Crotophaga ani*	X-22.75031; Y-43.72657
35	Butter Frog	*Leptodactylus latrans*	X-22.74569; Y-43.72458
36	Butter Frog	*Leptodactylus latrans*	X-22.75414; Y-43.72891
37	Cat-eyed Snake	*Corallus hortulanus*	X-22.74258; Y-43.72300
38	Snake	Ophidia	X-22.71069; Y-43.70052
39	Greater Ani	*Phacellodomus rufifrons*	X-22.72569; Y-43.71304
40	Opossum	*Didelphis aurita*	X-22.72253; Y-22.72253
41	Black Curassow	*Crotophaga ani*	X-22.70687; Y-43.69870
42	Dove	*Columbina talpacoti*	X-22.75043; Y-43.72707
43	Bat	Phyllostomidae	X-22.70233; Y-43.69580
44	Opossum	*Didelphis aurita*	X-22.75058; Y-43.72704
45	Potoo	*Hydropsalis longirostris*	X-22.70203; Y-43.69562
46	Bat	Phyllostomidae	X-22.74855; Y-43.72602
47	White-headed Curassow	*Guira guira*	X-22.72613; Y-43.71293
48	Opossum	*Didelphis aurita*	X-22.72588; Y-43.71271
49	House Wren	*Troglodytes musculus*	X-22.69288; Y-43.68754
50	Bat	Phyllostomidae	X-22.69325; Y-43.68795
51	House Wren	*Troglodytes musculus*	X-22.74096; Y-43.72172
52	White-winged Dove	*Leptotila verreaux*	X-22.74343; Y-43.72296
53	Green Kingfisher	*Didelphis aurita*	X-22.70731; Y-43.69852
54	Southern Lapwing	*Chloroceryle americana*	X-22.74626; Y-43.72488
55	Eastern Screech Owl	*Vanellus chilensis*	X-22.72880; Y-43.71518

**Table 2:** Following the adopted methodology, the identified results of the mycobiota over the one-year period are presented below.

**Sample**	**Animal**	**Mycobiota**	**Sample collected from**
1	Opossum	*Curvularia* sp*., Cladosporidium* sp. and *Aspergillus nidulans*	Fur
2	Bat	Sterile hyphae	Fur
3	Common Waxbill	Order Mucorales and *Aspergillus fumigatus*	Feathers
4	Crab-eating Raccoon	*Fusarium* sp.	Fur
5	Bat	*Rhyzopus* sp.	Feathers
6	Capybara	Order Mucorales	Fur
7	Bat	Order Mucorales	Fur
8	Bat	Order Mucorales	Fur
9	Caracara	*Rhyzopus* sp.	Feathers
10	Crab-eating Fox	Order Mucorales	Fur
11	Capybara	*Cladosporium* sp.	Fur
12	Black Vulture	Order Mucorales	Feathers
13	Burrowing Owl	Order Mucorales	Feathers
14	Opossum	*Nigrospora* sp.	Fur
15	Bat	Order Mucorales; *Aspergillus* sp.; dematiaceous	Fur
16	Bat	*Pestalotia* sp	Fur
17	Hummingbird	*Cladosporium* sp; *Fusarium* sp.	Feathers
18	Bat	*Cladosporium* sp.	Fur
19	Yellow-legged Rail	*Fusarium* sp.	Feathers
20	Butter Frog	*Basidiobolus* sp.	Scales
21	Snake	*Aspergillus* sp; *Fusarium* sp.*;*	Feathers
22	Eastern Screech Owl	*Curvularia* sp*; Fonsecaea* sp.	Skin
23	Opossum	*Aspergillus* sp*; Fusarium* sp.*;*	Fur
24	Crab-eating Fox	*Asspergillus niger; Chrysonilia* sp*;* dematiaceous;	Fur
25	Crab-eating Fox	*Chrysonilia* sp. e dematiaceous;	Fur
26	Capybara	*Mucor* sp.	Fur
27	Opossum	*Curvularia* sp.	Fur
28	Neotropical porcupine	dematiaceous	Spines
29	Marmoset	*Acremonium* sp. and *Cladosporium* sp.	Fur
30	Opossum	*Fusarium* sp.	Fur
31	Crab-eating Raccoon	Mucorales; *Penicillium* sp.	Fur
32	Neotropical porcupine	*Mucorales*	Fur
33	Opossum	*Mucorales*	Fur
34	Black Curassow	*Fusarium* sp.	Feathers
35	Butter Frog	*Aspergillus ochraceus*	Swab
36	Butter Frog	bacterial growth	Swab
37	Cat-eyed Snake	Mucorales; *Aspergillus* sp.	Swab
38	Snake	*Curvularia* sp.	Swab
39	Greater Ani	bacterial growth	Feathers
40	Opossum	*Fusarium* sp.	Fur
41	Black Curassow	Mucorales	Feathers
42	Dove	*Aspergillus niger*	Feathers
43	Bat	*Aspergillus niger*	Fur
44	Opossum	*Aspergillus niger*	Fur
45	Potoo	*Mucorales*	Feathers
46	Bat	*Mucorales*	Fur
47	White-headed Curassow	*Aspergillus niger*	Feathers
48	Opossum	*Mucorales*	Fur
48	House Wren	*Aspergillus niger*	Feathers
50	Bat	*Aspergillus niger*	Fur
51	Wren	*Aspergillus niger*	Feathers
52	White-winged Dove	*Mucorales*	Feathers
53	Green Kingfisher	*Aspergillus* sp.	Feathers
54	Southern Lapwing	*Aspergillus niger*	Feathers
55	Eastern Screech Owl	*Aspergillus niger; Aspergillus* sp.*; Fusarium* sp.*; Mucorales*	Feathers

## Discussion

According to Milli & Passamani (2006), in their study on the impact of wildlife roadkills along Highway ES-259 in 2004, the spatial distribution of collisions did not reveal a specific hotspot of higher incidence. Similarly, the current study did not identify a concentrated area of roadkill events along the defined section. Another point of convergence is that the highest number of roadkill incidents occurred in regions with predominant urban activity. Future studies may open doors to elucidating whether these fungi can be used as an ecological alternative in the composting of animal carcasses and, in this way, accelerate the decomposition of the carcass and its reintegration into the environment.

Among the genera identified, *Aspergillus* sp. was the most prevalent. Although this genus predominantly comprises saprophytic fungi, it can also act as an etiological agent in plants and animals, causing various diseases (De Hoog et al., 2021). *Aspergillus* is frequently found in soil, plant debris, wood, excrement, and hair, among other substrates (De Hoog et al., 2021). These characteristics support its development in cadaveric environments. Species of *Aspergillus* have been reported to cause infections in wild mammals and birds (Echenique et al., 2016), and such species were identified in samples collected from carcass remains in this study.

*Fusarium* is a genus that includes saprophytic fungi commonly found in soil, water, and various plants, where they often act as phytopathogens (De Hoog et al., 2021). In the present study, it was isolated from decomposing carcasses and may play a role in the decomposition of animal organic matter.

Among the group of dematiaceous fungi identified were *Cladosporium* sp., *Nigrospora* sp., and *Curvularia* sp. *Cladosporium* was the second most prevalent genus. These filamentous, melanized fungi are common in hot and humid climates and are known to cause phaeohyphomycosis—an opportunistic fungal infection that can be cutaneous, subcutaneous, or systemic—in animals (Jarrah et al., 2017; Samson et al., 2019; De Hoog et al., 2021). According to Menezes et al. (2017), *Cladosporium* is frequently isolated as a contaminant. However, in this study, it was not identified as such, as the sample was inoculated in triplicate (from the same sample) and the fungus grew consistently on all plates. Fungi of the genus *Nigrospora*, belonging to the phylum Ascomycota, are widely distributed in tropical and subtropical environments, especially in plant decomposition. These filamentous and melanized fungi may behave as opportunistic pathogens, particularly in immunocompromised animals, and can cause phaeohyphomycosis (Aquino & de Moura e Lima, 2022). In this study, *Nigrospora* was isolated from the carcass of an opossum and may thus be considered a cadaveric decomposer.

The genus *Curvularia* comprises a diverse group of fungi that can be saprophytic, endophytic, and/or pathogenic in plants, animals, and humans (Iturrieta-González et al., 2020). Species from this genus have been reported to cause both superficial and deep infections, including respiratory infections and even cerebral phaeohyphomycosis (De Hoog et al., 2021). In this study, *Curvularia* was isolated from the carcass remains of a mammal and an amphibian, suggesting its role as a decomposer of animal tissues.

*Basidiobolus* spp. are both parasitic and saprobic, having been found in insects, wood lice, decaying vegetation and in the faeces of amphibians and reptiles. (Shaikh et al., 2016). In this study, it was isolated from the decomposed carcass of an ophidian, supporting its association with the intestinal flora of these animals.

There is some disagreement in the literature regarding the classification of *Pestalotia* sp., which is often referred to as *Pestalotiopsis* sp., with the former sometimes considered a synonym of the latter (Cardoso et al., 2003). This genus includes phytopathogenic fungi that typically cause leaf spot disease, characterized by small necrotic lesions on leaves, stems, and fruits. However, in this study, *Pestalotia* sp. was identified for the first time as part of the mycobiota in the cadaveric decomposition of a bat. It is therefore important to determine whether its presence was due to plant material, gastric contents, soil contamination, or its role as a true decomposer.

It's difficult to separate the roles of environmental fungi and decomposers, as they overlap. The decomposition of animals and plants generates organic matter that returns to the environment and leaf litter, contributing to the Earth's carbon cycle.

## Conclusion

A great diversity of fungi was identified in all samples of cadaveric remains collected from carcasses found throughout the period, with different species belonging to more than ten fungal genera, including *Mucor* sp. and *Rhizopus* sp. (Order Mucorales), *Aspergillus* sp., *Cladosporium* sp., and *Fusarium* sp. This is the first study to analyze the frequency of roadkill among wild animals along the BR-493 highway in Seropédica, Rio de Janeiro, Brazil, and to isolate fungi from their remains. This is a unique, multidisciplinary study. The list of species documented along the highway over a two-year period contributes to studies of the impact of urbanization on wildlife and how to mitigate its effects. The list of fungi isolated from the remains contributes to the study of forensic mycology without the use of laboratory animals or interfering with wildlife in the region. More researchs should make controlled results, using carcasses in different stages of decomposition, on the growth kinetics of the identified fungal genera. Further studies should be carried out in the area, comparing the relative frequencies of fungi isolated by species of animal run over over a longer period of time, and in other areas of Brazil that also have highways or construction projects impacting wildlife.
